# Transcriptome profiling of the demosponge *Amphimedon queenslandica* reveals genome-wide events that accompany major life cycle transitions

**DOI:** 10.1186/1471-2164-13-209

**Published:** 2012-05-30

**Authors:** Cecilia Conaco, Pierre Neveu, Hongjun Zhou, Mary Luz Arcila, Sandie M Degnan, Bernard M Degnan, Kenneth S Kosik

**Affiliations:** 1 Neuroscience Research Institute and Department of Molecular, Cellular and Developmental Biology, University of California, Santa Barbara, CA, 93106, USA; 2 Kavli Institute for Theoretical Physics, University of California, Santa Barbara, CA, 93106, USA; 3 Centre for Marine Science, School of Biological Sciences, University of Queensland, Brisbane, QLD, 4072, Australia; 4 Present address: Cell Biology and Biophysics Unit, European Molecular Biology Laboratory, Heidelberg, 69117, Germany

## Abstract

**Background:**

The biphasic life cycle with pelagic larva and benthic adult stages is widely observed in the animal kingdom, including the Porifera (sponges), which are the earliest branching metazoans. The demosponge, *Amphimedon queenslandica*, undergoes metamorphosis from a free-swimming larva into a sessile adult that bears no morphological resemblance to other animals. While the genome of *A. queenslandica* contains an extensive repertoire of genes very similar to that of complex bilaterians, it is as yet unclear how this is drawn upon to coordinate changing morphological features and ecological demands throughout the sponge life cycle.

**Results:**

To identify genome-wide events that accompany the pelagobenthic transition in *A. queenslandica*, we compared global gene expression profiles at four key developmental stages by sequencing the poly(A) transcriptome using SOLiD technology. Large-scale changes in transcription were observed as sponge larvae settled on the benthos and began metamorphosis. Although previous systematics suggest that the only clear homology between Porifera and other animals is in the embryonic and larval stages, we observed extensive use of genes involved in metazoan-associated cellular processes throughout the sponge life cycle. Sponge-specific transcripts are not over-represented in the morphologically distinct adult; rather, many genes that encode typical metazoan features, such as cell adhesion and immunity, are upregulated. Our analysis further revealed gene families with candidate roles in competence, settlement, and metamorphosis in the sponge, including transcription factors, G-protein coupled receptors and other signaling molecules.

**Conclusions:**

This first genome-wide study of the developmental transcriptome in an early branching metazoan highlights major transcriptional events that accompany the pelagobenthic transition and point to a network of regulatory mechanisms that coordinate changes in morphology with shifting environmental demands. Metazoan developmental and structural gene orthologs are well-integrated into the expression profiles at every stage of sponge development, including the adult. The utilization of genes involved in metazoan-associated processes throughout sponge development emphasizes the potential of the genome of the last common ancestor of animals to generate phenotypic complexity.

## Background

For many animals, the genome controls the construction and function of two or more discrete life forms, each with its own morphological, physiological, behavioral and ecological characteristics. These life cycles, which typically include larval and adult phases, require the successive unfolding of morphogenetic programs at embryogenesis and metamorphosis. The pelagobenthic life cycle, in which a pelagic, microscopic larva metamorphoses into a large, benthic adult, is one common variation. An inherent feature of the pelagobenthic life cycle is the settlement of the planktonic larva, usually in response to an environmental cue, and its subsequent metamorphosis into the benthic form. This major ecological transition dictates the likelihood of future reproductive success because adults usually are sessile or sedentary, and therefore, larvae must settle at a distance from conspecifics to allow subsequent effective matings. Before settling and metamorphosing, the larvae must first develop the capacity, or competence, to detect and respond to benthic environmental cues typically associated with post-settlement survival and reproduction (e.g. signals from food items and conspecific adults) [1].

During development, a single gene inventory is drawn upon to implement the vast morphological and ecological changes associated with metamorphosis from one body form into another. Gene expression studies at developmental transitions, therefore, offer a window into the genetic framework that corresponds to distinct morphological features and ecological demands. Genome-wide activity as the animal prepares and moves from the pelagic to the benthic phase has been studied in just a few animals, including ascidians, gastropods and corals [2-8]. These gene expression studies of metamorphosis in animals from diverse phyla have uncovered few conserved features of this essential life cycle transition.

**Figure 1:**
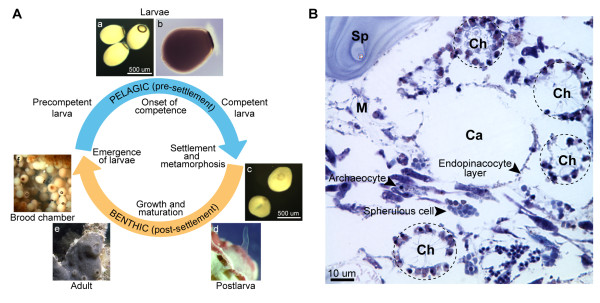
**The life cycle of the demosponge *Amphimedon queenslandica*.** (**A**) Precompetent larvae emerge from maternal brood chambers (f) and then swim in the water column for a minimum of 4 hours before they develop competence to settle and initiate metamorphosis (a-b). Upon settling, the larva adopts a flattened morphology as it metamorphoses into a postlarva (c), which after 3 days of development displays most of the hallmarks of the adult body plan, including an aquiferous system with canals, choanocyte chambers and oscula (d). This settled postlarva will grow and mature into an adult sponge (e) with brood chambers housing embryos and larvae (f). (**B**) Cellular organization in the *A. queenslandica* adult. The interior of the adult sponge is an extensive aquiferous system with water canals (Ca) embedded in a collagenous mesohyl (M) and supported by siliceous spicules (Sp). Pinacocytes form the exopinacoderm and endopinacoderm, which line external surfaces and internal canals, respectively. Water canals are surrounded by choanocyte chambers (Ch) lined with flagellated choanocytes that filter surrounding seawater for food. Other cells in the sponge, such as amoeboid stem cell-like archaeocytes and vacuole-filled spherulous cells, are interspersed in the mesohyl. Sponge tissue section was stained with toluidine blue.

In the demosponge, *Amphimedon queenslandica,* the pelagobenthic life cycle starts with the emergence of larvae from brood chambers of the adult (Figure 1A). Larvae are composed of several cell types organized into three distinct layers—an inner cell mass, a subepithelial layer, and a ciliated outer epithelium—and is patterned along the anterior-posterior swimming axis [9]. Formation and patterning of the larva appears to require a suite of conserved developmental transcription factors and signaling pathways [10-12]. Larvae require at least four hours of further development in the plankton before they attain metamorphic competence and become able to respond to environmental inductive cues and initiate settlement and metamorphosis [13]. During this time, larvae display negative phototactic behavior that is mediated by a ring of long-ciliated, pigmented cells at the posterior pole [14]. The surface of larvae possesses sensory cells expressing a pro-neural transcription factor [15] and synaptic gene orthologs [16] that are suspected to be involved in the detection of inductive cues signaling the presence of appropriate settlement sites [17]. After settlement, the sponge undergoes metamorphic transition, when extensive reorganization of undifferentiated cells and trans-differentiation of functional larval cells into adult structures occurs [9,18,19]. There is no evidence of extensive autolysis during sponge metamorphosis [9]. The adult sponge bears no morphological resemblance to other animals [20]; it is characterized by a few distinct cell types loosely embedded in a collagen-based mesohyl supported by a siliceous spicule skeleton (Figure 1B). The cells in the adult form an extensive aquiferous system that efficiently filters seawater for food in the form of bacteria and other particulate matter [21,22]. 

A strong theme has emerged among the growing number of complete genomes from extant representatives of the earliest diverging metazoans, including the sea anemone, *Nematostella vectensis*, and the hydra, *Hydra magnipapillata*, the placozoan, *Trichoplax adhaerens*, and as studied here, the haplosclerid demosponge, *A. queenslandica*, that despite their relatively simple body plans they possess a gene repertoire that is comparable to that of the bilateria [23-26]. For example, sequencing of the *A. queenslandica* genome has revealed a surprisingly complex complement of genes important for metazoan multicellularity, including those with known roles in cell adhesion, self and non-self distinction, immunity, controlled proliferation, cell death, and differentiation [23]. However, it is yet unknown which sets of genes are expressed at each developmental stage to coordinate the changing morphological features and ecological demands throughout the sponge life cycle. Whether the adult sponge, which lacks discernable features that readily link it to other animals, utilizes the bilaterian gene set or exploits uniquely Poriferan genes to implement its distinct morphology remains undetermined.

With the rudiments of the animal gene inventory in place at the time the earliest metazoans diverged, a large portion of evolutionary innovation thus has likely arisen by modification of gene regulatory networks [27]. Given the early-branching position of Porifera and their success in widely speciating through diverse eco-niches while maintaining a simple body plan for the past 580 million years [28], these organisms offer a unique opportunity to explore the origin and evolution of metazoan gene networks and the biphasic lifestyle. Our global study of the *A. queenslandica* transcriptome highlights the genomic events that accompany the transition from pelagic larvae to the benthic adult stage and point to an intricate network of regulatory mechanisms that coordinate the changes experienced by the sponge during the pelagobenthic transition. This study also reveals gene families with potential roles in competence, settlement, and metamorphosis in the sponge, including transcription factors, G-protein coupled receptors and other signaling molecules.

## Results

### Deep sequencing of the sponge poly(A) RNA transcriptome

**Figure 2:**
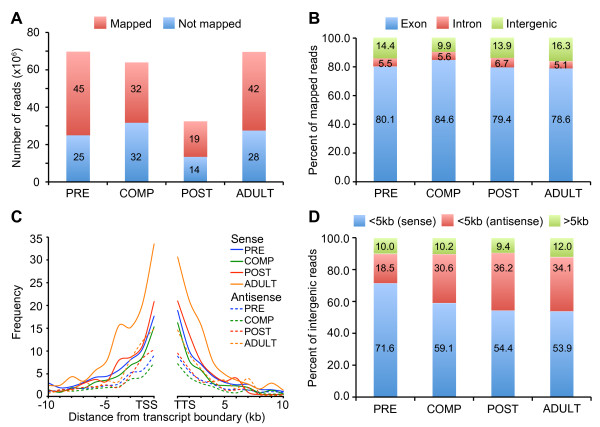
**Overview of read mapping to the *A. queenslandica* genome.** (**A**) Number of reads uniquely mapped to the genome for each developmental stage sequenced. (**B**) Percent of reads mapping to exons and introns of predicted gene models (Aqu1), and to intergenic regions. (**C**) Distribution of intergenic reads in relation to distance from the 5’ end (transcription start site; TSS) or 3’ end (transcription termination site; TTS) of predicted gene models. The average number of reads per 10 million mapped reads per sample was summed in 1 kb segments from the boundaries of annotated genes. (**D**) Percent of intergenic reads that map in proximity to predicted genes.

The transcriptome of the demosponge *A. queenslandica* was profiled at four stages of its life cycle spanning the pelagobenthic transition (Figure 1A). Sequencing libraries were constructed from poly(A)-enriched mRNA to eliminate sequences from prokaryotic symbionts usually associated with sponges [29]. Each stage was sequenced to a depth of 33–70 million reads (Figure 2A). On average, 58.3% of sequenced reads could be mapped to unique locations in the *A. queenslandica* genome (19–45 million mapped reads). Observed mapping statistics are similar to those reported in previous RNA sequencing studies [30,31]. On average, 80.7% of uniquely mapped reads were located in predicted exons, 5.7% in introns, and 13.6% in intergenic regions (Figure 2B). The majority of intergenic reads (90%) were located in proximity to annotated genes (Figure 2C-D). More than 50% of intergenic reads are oriented on the sense strand relative to neighboring genes and most likely represent the transcription boundaries of predicted sponge gene models, including 5’ and 3’ untranslated regions [32]. 

### Transcriptional complexity across the *A. queenslandica* life cycle

**Figure 3:**
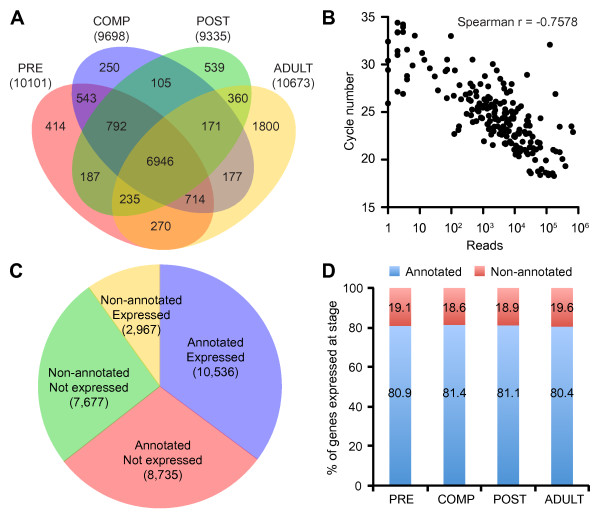
**Identification of expressed genes.** (**A**) Stage distribution of transcripts detected above the cutoff threshold of 64 reads. The total number of transcripts detected at each stage is in parentheses. PRE, precompetent; COMP, competent; POST, postlarvae; ADULT, adult stage. (**B**) Comparison of sequencing read counts and quantitative RT-PCR cycles for 50 selected transcripts in the four developmental stages. (**C**) The number of annotated versus non-annotated genes detected by sequencing. Gene annotations are based on alignment to known sequences in the UniProt database (e-value ≤ 1x10^-4^). Non-annotated genes have no significant matches in UniProt and may include potential ‘sponge-specific’ genes. Only 28% of non-annotated genes were found to be expressed compared to 55% of annotated genes. (**D**) Similar percentages of annotated and non-annotated sponge genes are expressed at each developmental stage.

Reads mapping to the *A. queenslandica* genome were normalized to sequencing depth and transcript expression was determined by the number of normalized reads associated with the exons of each predicted gene model (Additional file 1). Overall, transcriptome sequencing detected 21,743 genes with at least one read (72.5% of predicted gene models) and 13,503 genes (45% of predicted gene models) were confidently detected above the cutoff threshold of 64 reads in at least one of the stages profiled. The transcript detection cutoff was determined to be the minimal read count above which all transcripts are detected in two independently prepared libraries from the same starting RNA sample (Additional file 1). Approximately 10,000 genes (~33%) were detected at each stage of the sponge life cycle, with 6,946 genes detected above threshold in all stages (Figure 3A). The number of genes detected in the four stages of the sponge life cycle included in this study approaches the ~11,000 genes reported in a transcriptome profiling study of mouse stem cells using the same sequencing platform [30]. To determine if sequencing read counts obtained from a single pool of individuals can be recapitulated in biological replicates, we performed quantitative RT-PCR (qPCR) on three different pools of sponge material for each developmental stage. qPCR cycles correlated well with sequencing read counts normalized to total mapped reads (Spearman r = −0.7578; Figure 3B). For 40 out of 50 genes tested, the two methods detected similar expression trends across development (Pearson r ≥ 0.70; Additional file 2). Stage-specific differences in expression greater than four-fold as detected by sequencing were usually also detected as significant by qPCR (p < 0.01, ANOVA). Sequencing and quantitative qPCR have different dynamic ranges of detection that, along with variable qPCR primer efficiencies, may contribute to the differences observed between techniques. Correlation between the two methods of transcript quantitation are likely to improve as the sponge genome assembly and gene definitions are further refined. 

### Expression of annotated and non-annotated sponge genes

*A. queenslandica* genes without identifiable homologs in other organisms are also likely to contribute to environmental adaptations, as was reported in the microcrustacean *Daphnia pulex*, a genome in which more than a third of genes lack detectable homologs in any other available proteome, and are the most responsive genes to ecological challenges [33]. About 36% (10,645 out of 29,915 genes) of *A. queenslandica* protein coding genes have no identifiable homologs in other species, as determined by alignment to proteins in the UniProt database [34]. 1,536 of these genes without identifiable homologs have recognizable PFAM domains. Expressed sequence tag analysis in two other demosponges, *Suberites domuncula* and *Lubomirskia baicalensis*, revealed a similar percentage of genes that could not be assigned to any known function based on homology to other species [35]. The set of non-annotated sequences is likely to include novel ‘sponge-specific’ genes, although some erroneous gene models may also be present [23]. This is supported by the finding that only 28% of non-annotated genes (2,968 out of 10,644 genes) were robustly expressed above the cutoff threshold of 64 reads compared to 55% (10,535 out of 19,271 genes) of annotated genes (Figure 3C). Sponge-specific genes also tend to have lower expression levels than annotated genes, however, they exhibit the same overall patterns of variation across development (Figure 3D, Additional file 3).

### Expression trends across development

**Figure 4:**
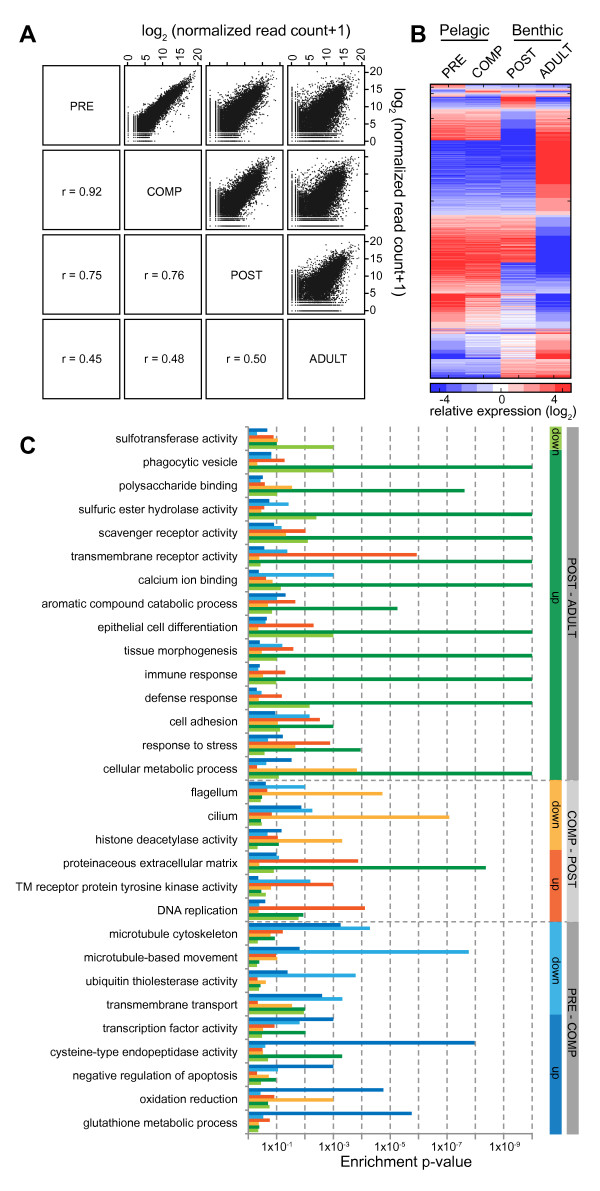
**Transcriptome profile of the developing sponge.** (**A**) Correlation of expression for 13,503 genes detected above the cutoff threshold across different stages of development (r, Pearson correlation coefficient). The x and y axes are log_2_ of read counts +1 normalized to the total number of mapped reads. (**B**) Expression profile for genes that are differentially expressed during development. Data for 4,677 genes with greater than four-fold change in expression in one stage compared to the other three stages is shown (p < 0.05, two-tailed *t*-test). Each row represents data for one gene. Pelagic stages include precompetent (PRE) and competent (COMP) larvae; benthic stages include postlarvae (POST) and adult (ADULT). (**C**) Gene ontology (GO) analysis for gene groups up- or downregulated by greater than four-fold between successive stages. Enrichment p-values for selected terms are shown. GO terms are grouped by stage transition (indicated by gray bars to the right) while colored bars indicate the direction of change (up or downregulation) at each transition.

Comparison of global gene expression across the pelagobenthic transition shows that the early swimming stages have very similar transcription profiles, although significant differences occur in expression of genes that presumably are related to the development of metamorphic competence. In contrast, as larvae metamorphose into the postlarval form, large-scale changes in transcription occur (Figure 4A). To determine primary expression trends in the transcriptome profile, we compared the expression of each transcript at each stage versus its mean in the other three stages (stage-specific) or between the two larval stages and the two post-settlement stages (settlement-specific). We filtered out transcripts for which expression varied by less than four-fold between the sets of stages being compared and for which stage expression did not differ significantly from the mean of the other stages (p < 0.05, two-tailed *t*-test). The cutoff for differential expression was chosen based on the largest fold change observed within technical replicates. Out of all the genes that were detected by sequencing, 4,677 (21.5%) exhibited either stage- or settlement-specific expression (Figure 4B). Lowering the cutoff to genes with a two-fold change results in the detection of 5,984 genes with variable expression across development. A majority of genes that are differentially expressed by greater than four-fold are found at relatively higher levels in either the pelagic larval (1,943 pre-settlement) or in the benthic stages (1,043 post-settlement). Many genes also displayed significantly different expression in the benthic postlarvae (196 upregulated, 496 downregulated) and benthic adult stages (1,187 upregulated, 756 downregulated). Of these robustly changing transcripts, 26% (1,233 genes) are non-annotated and are potentially sponge-specific genes.

A limitation of the previous method is that it only detects stage-specific changes that are robust across all life cycle stages and overlooks genes with variable expression patterns. Thus, to identify genes that are differentially expressed at stage transitions, we followed the expression trajectory of every gene by performing pairwise comparisons of their expression between successive stages. Genes were categorized as upregulated, downregulated, or unchanged. From this analysis, we identified 8,219 genes (37.8%) that exhibited greater than four-fold change in expression between any two successive stages, of which 2,178 (26.5%) are sponge-specific (Additional file 6). If the cutoff threshold is lowered to two-fold, the number of differentially expressed genes increases to 11,888 with 2,759 (23%) sponge-specific transcripts ( Additional file 7). At the transition from the precompetent to the competent stage, a total of 491 genes were upregulated at least four-fold while 804 were downregulated. As settled postlarvae began metamorphosis into the juvenile body plan, 1,582 genes were upregulated and 2,204 were downregulated. Maturation from postlarval juvenile to adult was accompanied by the upregulation of 3,848 genes and downregulation of 1,980 genes. It should be noted, however, that because the competent and postlarval samples were sequenced at lesser depth than the other stages, genes expressed at low abundance in these samples may not be detected above the cutoff threshold. Mapped reads in the range of 20 million, as is the case for the postlarval sample, allow a close estimate of expression level for approximately 70% of low abundance genes [31]. Thus, the number of genes that appear to be downregulated at the precompetent-competent and at the competent-postlarva transition, as well as genes that are upregulated at the postlarva-adult transition may be overestimated.

### Larval stage expression profiles and determinants of competence

Functionally related *A. queenslandica* genes were identified by Gene Ontology (GO) [36] annotation based on their best sequence similarity match to proteins in the UniProt database [34]. GO annotation enrichment analysis was performed on groups of genes that are differentially expressed by greater than four-fold at specific developmental transitions to determine the processes that are up- or downregulated at particular ontogenetic stages (Figure 4C, Additional file 8). Although the set of genes that change in expression between each stage encodes diverse cellular functions, we were able to observe enrichment of specific functional categories within gene groups with similar expression trends. GO analysis on the set of genes that are differentially expressed by greater than two-fold between successive stages revealed enrichment of a comparable set of GO categories (Additional file 8). Further analysis of available PANTHER [37] annotations for the same gene groups yielded enrichment of specific domains or enzymatic activities that fall under the more general categories seen by GO analysis (Additional file 9).

As larvae attain metamorphic competence (the capacity to initiate metamorphosis by detecting and responding to inductive environmental cues), genes encoding dyneins, kinesins, and other proteins involved in microtubule cytoskeleton assembly and microtubule dynamics become downregulated (Figure 4C). Cilia and flagellar components are further downregulated at the transition to postlarva when cilia appear to be resorbed and major morphogenic changes begin. Ubiquitin thiolesterase genes, which protect proteins from proteasome degradation, are also downregulated in the competent stage, suggesting that protein turnover may be an important mechanism regulating this transition.

In competent larvae, transcripts that encode genes with potential protective functions, such as negative regulators of apoptosis (Bcl2, Bax), antioxidants (glutathione metabolism enzymes, superoxide dismutase, peroxiredoxin, catalase), and stress response proteins (Hsp70, Sap16, pirin, sequestosome) are upregulated (Figure 4C, Additional file 8 and Additional file 9). Genes that regulate transcription, including CCAAT-box binding proteins, CRE-binding proteins, bHLH factors, and homeobox proteins are also enriched. As expected for lecithotrophic larvae that rely on metabolism of yolk stores for energy generation, many genes involved in cellular respiration are found at their highest levels in pelagic larvae (Additional file 4). Furthermore, there is an enrichment of transcripts regulating oxidation and reduction, including mitochondrial genes such as glycerol-3-phosphate dehydrogenase, electron transfer flavoprotein-ubiquinone oxidoreductase, and glycine dehydrogenase, in the set of genes that are upregulated in competent larvae. A similar upregulation of lipases and mitochondrial genes has been observed in lecithotrophic coral planula [3,6].

### Genome-wide expression profiles from the pelagobenthic transition

The transition from the planktonic competent larval stage to the benthic postlarval stage results in extensive cellular transdifferentiation, proliferation and rearrangement, resulting in the appearance of most of the cell types associated with the adult body plan arranged into functional morphological units such as pinacoderm and choanocyte chambers [9,19]. We observed transcriptional changes consistent with these processes and the extensive intercellular signaling pathways that may regulate morphogenetic events during metamorphosis. For example, many transcripts encoding transmembrane receptors, kinases, and signal transduction factors are upregulated in the postlarva (Figure 4C, Additional file 8 and Additional file 9). We also observed an increase in the expression of genes involved in DNA replication, DNA recombination, and methyltransferase activity that occur in preparation for cell division. Extracellular matrix components that make up the sponge mesohyl are also upregulated at this stage. Furthermore, there appears to be an overall decrease in the requirement for energy production as evidenced by downregulation of many mitochondrial enzymes with oxidation-reduction activity.

### The adult transcriptome

Although previous systematics suggest that the only clear homology between Porifera and other animals was in the embryonic and larval stages [20], analysis of the *A. queenslandica* genome revealed the presence of many of the genes involved in metazoan-associated cellular processes [23]. Transcriptome analysis showed extensive use of these genes throughout the sponge life cycle, particularly at the adult stage, where the differentially upregulated gene set is enriched for functions that are characteristically metazoan, such as cell adhesion, immune response, tissue morphogenesis, cell proliferation, and apoptosis. Transcripts encoding transmembrane receptors, adhesion molecules, and extracellular matrix components (Figure 4C, Additional file 8 and Additional file 9) are widely expressed in sponge with marked enrichment in the adult (Additional file 4).

With transition to the adult stage, upregulated genes include extracellular matrix components required to form the mesohyl or gelatinous matrix that fills the space between the external pinacoderm and the internal choanoderm. Multiple sulfatases and metallopeptidase-like proteases that are upregulated in the adult may also have a role in extracellular matrix remodeling (Figure 4C). The reliance of the adult on phagocytosis of food particles from surrounding seawater is reflected in the transcriptome by an increase in the expression of scavenger receptors and polysaccharide binding molecules (lectins) that enable the sponge to differentiate between food bacteria and symbiotic bacteria [38-42]**.** These same molecules may mediate cell-to-cell adhesion and allorecognition in the sponge [39,43,44]. The adult sponge upregulates the expression of various catabolic enzymes, including lysosomal enzymes such as sulfatases and fucosidases that degrade glycoproteins, glycolipids, and sulfated proteoglycans, as well as cysteine-type endopeptidases that degrade proteins (Additional file 8 and Additional file 9). Other enzymes upregulated in the adult, including multi-copper oxidases and aromatic compound metabolizing enzymes, may be involved in the synthesis of secondary metabolites that allow sponges to select for or against specific types of microorganisms [29,45].

Notably, about 70% (2,568 out of 3,661 genes) of the genes that are upregulated at the transition from postlarva to adult exhibit an expression change on the order of ten-fold or greater, with many of the transcripts displaying either larval/postlarval- or adult-specific expression. Overall, we identified 703 transcripts exhibiting a greater than 100-fold expression differential between one stage and the other three stages (Additional file 10). The majority of these genes are enriched in the adult (640 genes). These include the PDZ domain-containing protein Nherf2, which regulates targeting and trafficking of receptors, ion channels, and other membrane proteins [46-48], and syntrophin gamma, a cytoplasmic peripheral membrane protein that binds to components of mechanosensitive sodium channels [49]. Ion channels, including amiloride-sensitive sodium channels are also upregulated in the adult, suggesting a possible role in water homeostasis or coordination of cellular contraction [50].

### Functional gene families associated with competence and metamorphosis

#### Developmental signaling pathways

Members of developmental signaling pathways were expressed at variable levels in all the stages that were sequenced, indicating that the same pathways involved in early embryonic patterning [10,11] may also have a role in morphogenetic changes occurring after larval settlement (Additional file 5). Several Wnt receptors, such as Frizzled and Low-density lipoprotein receptor-related protein (Lrp5/6), Wnt ligands, and inhibitory molecules (Apc, Sfrp, Invs), are upregulated after settlement in postlarva. The expression of Wnt pathway genes in the adult sponge suggests roles for these genes beyond setting up embryonic polarity and may extend to the formation of water canal openings in the exopinacoderm [51-53]. Molecules in the Notch and TGF-β signaling pathways were also detected in all stages, although the expression of ligands and receptors were not always coordinated. While genes in the Wnt pathway appear to be generally downregulated at the postlarval stage (p < 0.05, Fisher’s exact test), there is no stage-specific enrichment or depletion observed for the Notch and TGF-β pathways.

#### Transcriptional regulators

**Figure 5:**
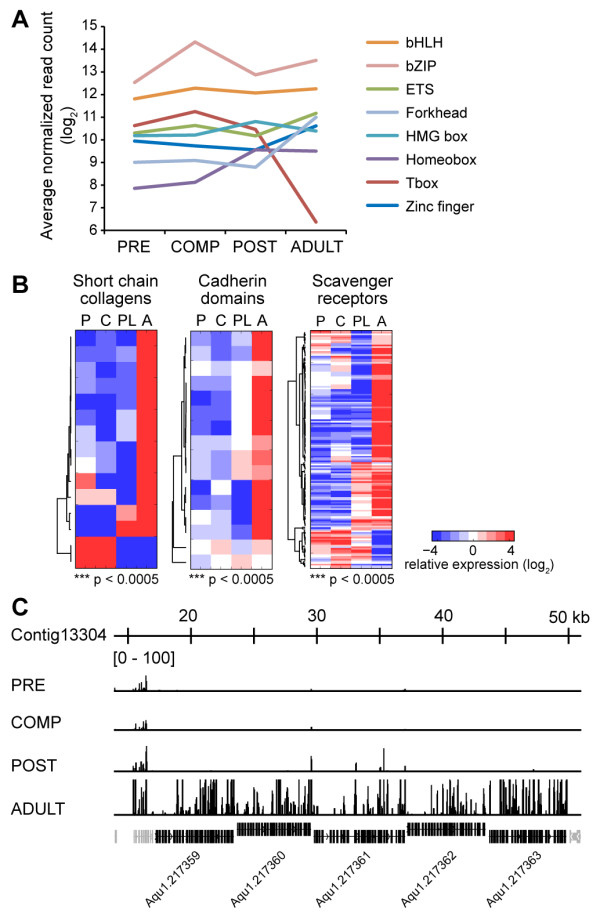
**Expression of transcription factors.** (**A**) Average normalized read counts for members of transcription factor families at four stages of *A. queenslandica* development. Genes were retrieved from previous studies [23,56,57]. (**B**) Gene families exhibiting coordinated gene expression. Short chain collagens, cadherin domain-containing, and scavenger receptor domain-containing gene families show specific upregulation at the adult stage. Heat maps show the relative expression of these genes across development (red, high; blue, low). Stage enrichment of genes was estimated using Fisher’s exact test (p-values shown). (**C**) Cluster of 5 scavenger receptor domain-containing genes that are expressed in the adult sponge. Reads mapping to this genomic locus in the different developmental stages are shown (reads normalized to 10 million, maximum peak height shown is 100 reads). Non-scavenger receptor genes in the cluster are shown in gray.

Transcription factors that emerged in the metazoan lineage are thought to have had an important role in development and differentiation of cell types [12]. Differential expression of transcription factors and transcriptional regulators suggest a crucial role in coordinating the transformations that occur during the pelagobenthic transition. About 86% of genes (151 out of 176 genes) bearing known transcription factor domains represented in the sponge genome were also detected in the sponge transcriptome (Figure 5A, Additional file 4). Members of the bZIP and Tbox families are enriched in competent larvae and may be involved in regulating the expression of genes necessary for settlement (p < 0.05, Fisher’s exact test; Additional file 5). However, despite upregulation of transcription factor expression, the total mRNA transcripts in competent larvae did not change dramatically from the precompetent stage, as evidenced by the well-correlated transcription profiles of the two stages (Figure 4A). This observation suggests that other regulatory proteins present in the pelagic larvae are providing an additional layer of control over gene expression. For example, genes encoding proteins similar to the repressors Ncor, Sin3, Tbl1xr, Phf, and Nacc, show a trend toward downregulation during the transition from larva to adult. Chromatin modifiers also show differential expression in pelagic versus benthic stages. While histone acetylation genes are found at lower levels (Additional file 4) and histone deacetylases (sirtuins) are highly expressed in competent larvae, the opposite is true after settlement (Figure 4C, Additional file 8 and Additional file 9). Thus, competent sponge larvae appear to be poised for rapid and widespread transcription upon settlement, with multiple mechanisms in place to control changes in gene expression. In the adult sponge, which displays a dramatically different transcription profile from larvae, various transcription factor groups, including the zinc finger, forkhead, ETS, and homeobox domain-containing factors, as well as other transcriptional activators, are upregulated (p < 0.05, Fisher’s exact test). Contributing to regulation of gene expression in the benthic stages are DNA methylation and histone acetylation proteins (Additional file 4), as well as components of the small RNA machinery [54] and RNA binding proteins [55] that are upregulated upon settlement. 

Genes that are selectively expressed in the same developmental stage may be regulated by the same set of transcription factors. For example, short-chain collagens and members of the cadherin domain and scavenger receptor domain-containing gene families (Figure 5B), show coordinated upregulation at the adult stage (p < 0.0005, Fisher’s exact test). In the adult, transcriptionally active genomic loci with three or more scavenger receptor domain-containing genes are organized in tandem arrays and are potentially regulated through a shared cis-regulatory element (Figure 5C).

#### Photosensory system

Upon emerging from the brood chamber *A. queenslandica* larvae are light-sensitive but are initially indifferent to environmental signals that can induce metamorphosis. They need to swim in the water column for at least four hours before they are able to respond behaviorally to settlement cues associated with benthic coralline algae [13]. Physiological differences between younger, unreactive larvae and competent older larvae are likely to include the deployment of additional functional sensory systems that allow them to sense and react to the benthic environment. Several mechanisms, including photosensory molecules, ion channels, G-protein coupled receptors (GPCR) and kinases may be key to this response.

Studies in *A. queenslandica* and *S. domuncula* have shown that cryptochromes and bioluminescence proteins, whose transcripts are regulated by light exposure, play a role in the sponge photosensory system [58-60] and may mediate the negative phototactic response exhibited by larvae [14]. Transcripts for these genes are expressed in all stages of *A. queenslandica*, with an increase in the expression level of cryptochromes, luciferase, and luciferin binding protein in the competent stage (Additional file 4). Upregulation of genes involved in calcium-mediated signaling, including the luciferin regenerating enzyme, occurred in the same stage. Some ion channels were expressed highly in the pre-settlement stages, suggesting that ionic concentration may be a contributing factor for larval settlement, as has been observed in the sponge *Aplysilla*[61].

#### G-protein coupled receptors and kinases

**Figure 6:**
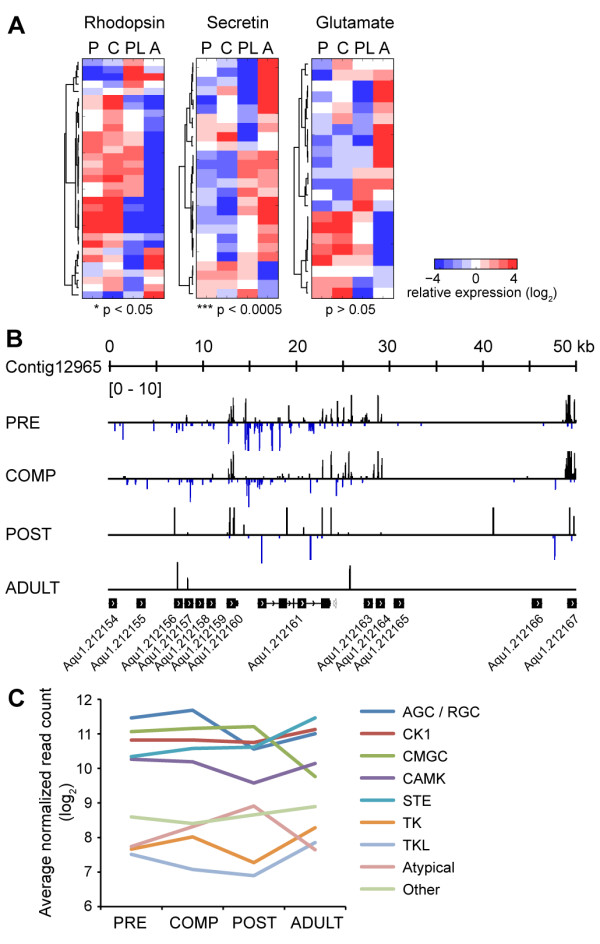
**Expression of G-protein coupled receptors and kinases.** (**A**) Relative expression of members of GPCR families defined in a previous study [23] across *A. queenslandica* developmental stages (red, high; blue, low). Stage enrichment of genes was estimated using Fisher’s exact test (p-values shown). (**B**) Gene contig with a cluster of 13 rhodopsin family GPCR genes (gene organization is indicated at the bottom). Reads mapping to the plus (black) and minus (blue) strands of this genomic locus in the four developmental stages are shown (reads normalized to 10 million, maximum peak height shown is 10 reads). A non-GPCR gene in the cluster is shown in gray. (**C**) Average normalized read counts for members of kinase classes defined in a previous study [23].

*A. queenslandica* has an extensive repertoire of GPCRs and kinases in its genome [23], suggesting that the organism possesses a sophisticated mechanism for monitoring and responding to its environment. The expression of a diverse family of GPCRs at different stages of the pelagobenthic transition may provide the mechanism by which larvae recognize inductive cues, such as amino acids, GABA analogs, or peptides, and identify suitable settlement sites [13]. While only 23% of predicted rhodopsin family receptor genes (33 out of 143 genes) were detected in at least one stage (Additional file 4), 25 genes were expressed at higher levels in pelagic larvae compared to the benthic stages (p < 0.05, Fisher’s exact test; Figure 6A), suggesting that this gene family may encode the chemoreceptors necessary for sensing inductive cues and identifying suitable settlement sites. Rhodopsin family genes are found in clusters on the genome; however, not all members of these clusters were detected (Figure 6B), perhaps due to low level expression or expression confined to just a few cells at specific developmental periods. In contrast, more than seventy percent of predicted metabotropic glutamate receptors (22 out of 25 genes) and secretin-family receptors (26 out of 36 genes) were detected in at least one of the stages sequenced (Additional file 4). Secretin-family receptors, some of which have hormone receptor, immunoglobulin, fibronectin, or EGF domains at their N-termini and are more similar to adhesion GPCRs [62], exhibit increased expression in the adult where they may be important for intercellular adhesion, environmental monitoring, or as components of the sponge immune response (p < 0.0005, Fisher’s exact test; Figure 6A). The metabotropic glutamate receptor family does not show stage-specific enrichment. Instead, this family appears to be divided into two major groups with upregulated expression in either the larval stages or in the adult. The members of this GPCR family that are highly expressed in the adult may have a role in coordinating cellular contractions in response to small molecules, a phenomenon that has been observed in *Ephydatia muelleri*[63], or in regulating the uptake of dissolved organic matter from seawater, which has been demonstrated in the calcisponge *Leucandra aspera*[64]**.**

The *A. queenslandica* genome encodes a wide array of kinases [23], a majority of which were detectable in all stages sampled (Additional file 4, Additional file 5). While we observed an increase in transcript expression for members of most kinase classes as the larvae mature into adults, the AGC/RGC and CAMK classes have higher average read counts in pelagic larvae (Figure 6C).

### Expression of genes found in bilaterian cell types

#### Epithelial genes

**Figure 7:**
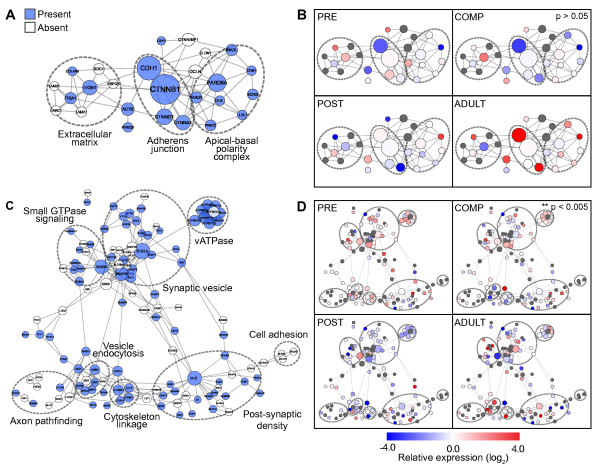
**Expression of epithelial and synaptic gene homologs.** Interaction network of epithelial (**A**) and synaptic (**C**) genes showing homologs that are present in the *A. queenslandica* genome (blue circles). Genes with related functions are indicated by dotted circles. Sponge genes were retrieved by matching Aqu1 gene models to previously published sequences [23,69]. Protein interactions were obtained from STRING [70] and displayed using Cytoscape [71]. Relative expression of epithelial (**B**) and synaptic (**D**) gene homologs in the four developmental stages (red, high; blue, low; gray, no sponge homolog). Stage enrichment of genes was estimated using Fisher’s exact test (p-values shown).

Although *A. queenslandica* bears little morphological similarity to other animals, it possesses homologs of various genes known to be found in bilaterian cell types, such as epithelia and neurons. Despite the lack of true epithelium, sponges can have the appearance of an epithelial organization in that the pinacocytes, which separate mesohyl from the environment and line external surfaces and the aquiferous canals, and choanocytes, which line choanocyte chambers, form epithelial-like cell layers (Figure 1B). Sponges also possess extracellular matrix components, such as short-chain collagens and fibronectins, that could support an epithelium [65,66], although a basal lamina with type IV collagen has been observed only in homoscleromorph sponges [67]. Furthermore, in a freshwater sponge, the pinacoderm can function like a true epithelium by controlling the passage of small molecules and generating a transmembrane potential [68]. Indeed, *A. queenslandica* possesses homologs of epithelial polarity and adherens junction genes (Figure 7A) [69]. These genes are expressed at all stages of sponge development (Figure 7B). Although genes in the epithelial network as a whole show no significant stage-specific enrichment, some members of the apical-basal polarity complex, as well as cadherin-domain containing proteins and multiple short-chain collagens, are enriched in the adult (Figure 5B). 

#### Neuronal genes

Porifera does not possess neurons or recognizable synapses but homologs of neuronal post-synaptic genes have been identified in the sponge genome (Figure 7C) and have been shown to be expressed in the globular cells of the outer epithelial layer of sponge larvae [16,23]. In the absence of true synapses, the role of these genes must be distinct from the function of bilaterian homologs. Indeed, while we observed a general downregulation of synaptic gene expression at the postlarval stage (p < 0.005, Fisher’s exact test), the overall expression pattern reveals a lack of coordinated regulation between the different elements that might be expected to generate a functional synapse (Figure 7D). Members of complexes involved in regulating vesicle dynamics, such as vesicular ATPases, synaptic vesicle proteins, and presynaptic signaling molecules show relatively higher expression in precompetent and competent stages, where the processes of intracellular transport, membrane trafficking, and signaling may be needed for the maintenance of ciliated epithelia and for the rapid response to the environment required by a pelagic lifestyle [72].

## Discussion

Deep sequencing of the transcriptome of the demosponge, *A. queenslandica*, as it develops from a pelagic larva to a benthic adult, revealed genome-wide transcriptional events accompanying this major life history transition. While metamorphosis is widespread throughout the animal kingdom, it is thought to have evolved several times in metazoans, thus there are likely to be different regulatory mechanisms controlling metamorphosis in various taxa [1]. For example, the metamorphic transition in marine invertebrates occurs rapidly compared to insects and other arthropods, making the period immediately after settlement, when the larva is most vulnerable, as brief as possible [73,74]. In this study, we discovered that competent *A. queenslandica* larvae retain a similar transcriptome profile to the precompetent larvae, but differentially express many transcription factors and regulators. This suggests that swimming sponge larvae already possess most of the gene products required at the pelagobenthic transition and are poised to enact rapid and widespread changes in gene expression upon settlement. One of the key mechanisms in this regulation may be the utilization of bivalent chromatin marks, as occurs in pluripotent cells [75]. Although the acquisition of metamorphic competence itself is not accompanied by much global change in transcription, the expression of select functional gene groups (e.g. transcription factors, rhodopsin family GPCRs, cryptochromes, calcium signaling proteins) does occur. The timely expression of competence genes allows the larva to disperse widely before settlement, navigate towards a suitable habitat, detect appropriate inductive cues, and adhere to its chosen substrate. GPCRs and membrane trafficking mechanisms that are expressed in the larval stages are candidate regulators of competence and metamorphosis, and indeed, exposure of precompetent larvae to an inductive cue results in habituation or a decrease in responsiveness that delays settlement [13].

For some species, the metamorphic transition can occur even with the inhibition of transcription and translation [1,73]. In contrast, we found that transformation of *A. queenslandica* from larva to the juvenile/adult is accompanied by a large change in transcription consistent with gross changes in morphology and the different environmental demands faced by free-swimming lecithotrophic larva and the sessile, filter-feeding adult. Pelagic larvae express genes required for metabolism of yolk stores and the maintenance of cilia and flagellar structures that allow them to remain in the water column. The decreased expression of genes involved in assembly of cilia/flagellar structures may be responsible, in part, for the decrease in swimming behavior that accompanies settlement on a substrate at the beginning of metamorphosis. The adult sponge, on the other hand, shows the hallmarks of a filter-feeding lifestyle, expressing genes that allow it to recognize food bacteria and to protect itself from potentially harmful microorganisms. Interestingly, metazoan developmental and structural gene orthologs are well-integrated into the expression profiles at every stage of sponge development. Rather than expressing mainly sponge-specific genes, the adult sponge expresses many genes that encode typical metazoan features, such as cell adhesion, cell differentiation, and immunity. No stage-selective expression was observed for the sponge-specific gene set (Figure 3, Additional file 3).

The genetic controls for metamorphosis are linked to evolutionarily ancient ‘toolkits’ that generate each staged body plan. In fact, the pelagobenthic transition is coordinated by a host of conserved developmental regulators (i.e. signaling pathway components and transcription factors) that are differentially expressed in larval and adult stages. For example, canonical Wnt signaling functions in the establishment of the anterior-posterior (AP) axis; the use of posterior Wnt signaling and anterior Wnt inhibition appears to be a unifying principle of the metazoan body plan development [76]. In *A. queenslandica*, Wnt expression is the earliest marker of asymmetry in the embryo; it becomes localized to the posterior pole as embryos begin to display AP polarity and this polar expression continues until the swimming larva stage [10,11]. Conservation is also apparent among those genes that have a likely role as downstream effectors of metamorphosis. Apoptosis, stress response, immunity, and calcium signaling are common functional groups upregulated during metamorphosis in various phyla [4,6,7,77]. Similar gene groups are expressed in the developing sponge, however, there is no evidence of extensive autolysis or apoptosis during sponge metamorphosis [9]. Like *Acropora millepora* coral larvae, *A. queenslandica* upregulates Bcl2, pirin, and catalase upon induction of metamorphosis [4]. Pirin and pirin-related genes are transcription cofactors that interact with nuclear factor I/CCAAT box transcription factor and forms a complex with Bcl-3 and NFκB to promote the transcription of anti-apoptotic genes in response to stress [78,79]. A decrease in the requirement for energy production, reflected in the downregulation of many mitochondrial enzymes with oxidation-reduction activity and of structural genes, is observed in the larvae of *A. queenslandica,* abalone (*Haliotis asinina*), and mollusc (*Aplysia californica*) as they undergo the metamorphic transition, as well as in fly larvae entering the pupal stage [5,7,80]. Thus, life cycle transitions may have deep homologies in their effector genes despite the widely different environmental settings in which these transitions unfold among organisms.

The current study was limited to the sequencing of single pooled samples from four well-defined developmental stages. Although each sample represents profiles from hundreds of larvae, this is a potential source of sampling bias since ensemble measurements may not account for greater variation in gene expression between individuals. The lack of biological replicates also limited our ability to detect genes with subtle changes over time. To minimize the effect of these biases, we estimated sampling error from technical replicates and used this as a benchmark to gauge the gene detection threshold and to set conservative criteria for the identification of differentially expressed genes. Similar gene ontology results for transcripts detected at two thresholds of differential expression, as well as validation by quantitative RT-PCR for a subset of genes and for biological replicates, provide some assurance that the changes we observed are robust. Inclusion of biological replicates in future studies, as well as increased sequencing depth, will allow more sensitive detection of differentially expressed genes. Further analysis of sponge gene expression at greater spatial and temporal resolution will allow the detection of genes with transient, cell type-specific, or stimulus-induced expression patterns. It is also important to note that some key regulatory events may be occurring at the post-transcriptional and post-translational levels that will not be observed by transcriptome sequencing. Expression studies, along with functional interrogation of genes identified here as potentially important for sponge development and metamorphosis, will ultimately provide a more complete picture of the pelagobenthic transition.

## Conclusions

Our findings point to a network of regulatory mechanisms that coordinate morphological changes with the ecological demands of the pelagobenthic transition. While differentially expressed groups of transcriptional regulators may mediate the widespread changes in gene expression that accompany the metamorphic transition, the identification of specific subsets of receptors that are upregulated during the period of competence suggests a potential link between morphogenesis and the environment. The vastly different transcriptome of the adult sponge, which expresses recently evolved metazoan genes involved in secondary metabolism, immune system, and stress response, most likely contribute to its ability to readily adapt to changing ecological conditions. The utilization of a conserved metazoan gene set throughout sponge development emphasizes the potential of the genome of the last common ancestor of animals to generate phenotypic complexity. This study provides a rich resource for the identification of mechanisms regulating major life cycle transitions, and will contribute to our understanding of sponge biology.

## Materials and methods

### Tissue samples

*A. queenslandica* were collected from Heron Island Reef, southern Great Barrier Reef, Queensland, Australia using a standardized protocol [81]. Precompetent larvae were collected not more than 3 hours after emergence from the brood chambers of the adult. Competent larvae were collected 6 hours after emergence. Postlarvae exhibiting a flattened juvenile body plan were collected after 2 days of settlement on glass coverslips. Due to the limited quantity of sponge material collected, we pooled about 1000 precompetent or competent larvae and 100 postlarvae to obtain sufficient RNA for library construction. Adult tissues were collected as a 5 mm core from apical to basal surface of individuals without brood chambers to avoid inclusion of early embryonic stages. All tissues were stored in RNAlater [Ambion] before processing.

### RNA extraction and poly(A) RNA purification

RNA from early larvae and postlarvae was extracted directly with Trizol [Invitrogen] following the manufacturer’s protocol. Adult samples were cleaned of macroscopic debris then ground in liquid nitrogen before RNA extraction with Trizol. Contaminating DNA was removed using the DNAfree kit [Ambion]. The poly(A) RNA fraction was enriched using the MicroPoly(A)Purist kit [Ambion] and ribosomal RNA was depleted using the RiboMinus Eukaryote kit [Invitrogen]. RNA quality was monitored using the Agilent Bioanalyzer RNA 6000 Pico Assay.

### Poly(A) RNA fragment library preparation and sequencing

Fragment libraries were prepared as previously described [30]. Briefly, approximately 250 ng of purified poly(A) RNA was subjected to 95°C until most of the RNA formed 50–200 nt fragments. First-strand cDNA synthesis was primed with a 3’ adapter-tagged random hexamer primer (sFDVhex) using SuperScript II reverse transcriptase [Invitrogen]. Second strand cDNA was then synthesized using a 5’ template switching adapter-tagged oligonucleotide (RDV-GGG). cDNA libraries were amplified using limited PCR cycles and fragments averaging 150 bp in length were purified. Libraries were quantified by PCR and fragment size was verified using the Agilent Bioanalyzer DNA 1000 Assay. 280 pg of each library was loaded onto beads by emulsion PCR. Approximately 40–80 million library beads were loaded on to separate slide sections and sequenced to 50 bases using the Applied Biosystems SOLiD V3 chemistry.

### Sequencing data analysis

Assembled sponge contigs and gene models (Aqu1) are publicly available [82]. Redundant transcripts for predicted gene models were filtered out retaining only the longest of overlapping transcripts originating from the same locus (29,915 unique genes). All 50 nucleotide reads were analyzed in color space using the SOLiD RNA Analysis Pipeline Tool [83]. Reads were aligned to sponge contigs using the ‘anchor-extend’ method as previously described [84]. Estimates of transcript expression were obtained by counting reads aligning to exons of predicted gene models. Sequencing data has been submitted to the NCBI Gene Expression Omnibus (GEO) as series GSE29978.

### Read normalization and identification of differentially expressed transcripts

Comparison of sequencing results from different libraries prepared from the same sample or libraries prepared from different biological samples showed that variable sequencing depths did not induce any detectable read count-dependent bias but rather affected read counts by a single scaling factor which is of the order of the difference in the total number of mapped reads. Read counts from different libraries were normalized by multiplying with a proportionality constant reflecting different sequencing depths to obtain read count distributions similar to the precompetent library, which was used as the reference sample. The distribution of reads in each sample after normalization is shown in Additional file 1. The transcript detection cutoff was determined to be the minimal read count above which all transcripts are detected in two independently prepared libraries from the same starting RNA sample (Additional file 1). Sampling statistics accounts for most of the differences between libraries in that read count differences between technical replicates of the same library, or between libraries derived from the same biological sample, scaled as √N irrespective of the read count N. The variation in read counts between two libraries was always smaller than 5√N for a given read count. The read count error equals 5√N/N, which decreases as the read count increases and is negligible for large read counts. Thus, reads with raw counts below 32 are likely to be detected in just one library out of two. To account for different sequencing depths, we set a normalized read count of 64 as the detection threshold (this number is equivalent to a threshold of 32 for the non-normalized postlarval sample, which had the lowest depth of sequencing).

In technical replicates of libraries derived from the same biological sample, the variation in expression for genes detected above 64 reads did not exceed three-fold. Fold change cutoffs as calculated based on read count error within technical replicates are 1.36, 1.68, and 2.25-fold for false calling rates of 5%, 1%, and 0.1%, respectively. Thus, we selected a four-fold change as a conservative criterion for differential expression. To identify genes with stage-specific expression across the pelagobenthic transition, we compared gene expression at each stage to the mean expression of the other three stages by performing a two-tailed *t*-test on the log of read counts and filtered out transcripts for which expression varied by less than four-fold. This method would filter out any gene that does not exhibit a strong stage-specific expression, particularly genes with dynamic expression across development. Therefore, to identify genes that exhibit differential expression between stages, we tracked the expression trajectory for each expressed gene in pair-wise comparisons between successive stages. Individual genes were categorized as either up or downregulated if their expression changed more than four-fold and were greater than the sampling error on the read count between stages. We also identified genes differentially expressed by two-fold between stages.

### Sequence alignment, annotation, and GO analysis

Sponge gene models were aligned to proteins in the UniProt database [34] using BLASTp [85] with an e-value cutoff ≤ 1x10^-4^. Genes were assigned the names and Gene Ontology (GO) [36] annotations of their best match. GO term enrichment p-values were estimated by comparing the enrichment of a particular GO term within a gene set to the enrichment distribution determined by re-sampling the set of all detected transcripts. GO enrichments are presented relative to the number of annotated genes and not of the entire genome, which includes about 30% genes without functional annotation. The statistical significance threshold was set to P < 0.05 (corrected for multiple hypothesis testing using the Benjamini-Hochberg method). Stage enrichment or depletion for genes in a specific functional group was determined using Fisher’s exact test on the number of genes at each stage that are detected above or below the upper 25% of its expression range across development.

### Quantitative real-time PCR

Primers for quantitative RT-PCR were designed to span exon junctions of predicted transcripts whenever possible to ensure specificity for target mRNAs (Additional file 11). All primers were synthesized by Integrated DNA Technologies (IDT). Total RNA for quantitative RT-PCR analysis was extracted from three separate pools of sponge material at each developmental stage. cDNA was synthesized using random primers and SuperScriptIII reverse transcriptase [Invitrogen] following the manufacturer’s protocol. Quantitative RT-PCR was performed using Power SYBR Green PCR master mix [Applied Biosystems] on an ABI 7500 Fast Real-Time PCR System. RNA expression was normalized to β-actin and quantified using the ΔΔCt method [86]. Variance of the pooled sample replicates was assessed using one-way ANOVA with Bonferroni’s post-test.

## Abbreviations

RNA : Ribonucleic acid; GO : Gene Ontology; GPCR : G-protein coupled receptor; qPCR : Quantitative RT-PCR.

## Competing interests

The authors declare that they have no competing interests.

## Authors’ contributions

CC, BMD, and KK designed the study; CC prepared libraries and performed experiments; MLA performed SOLiD sequencing; CC, PN, and HZ analyzed the data; CC, PN, BMD, SMD, and KSK wrote the manuscript. All authors read and approved the final manuscript.

## Supplementary Material

Additional file 1 **Figure S1. Read normalization and setting the transcript detection threshold.** (A) Distribution of transcript read counts after global normalization for sequencing depth. (B) Comparison of normalized read counts from two sequencing runs of the same library preparation and (C) comparison of normalized read counts from sequencing runs of two independent libraries made from the same RNA sample show that determination of transcript expression is reproducible above a cutoff threshold of 64 reads (red lines). Click here for file

Additional file 2 **Figure S2. Comparison of expression trends from sequencing and quantitative RT-PCR.** The expression of 50 transcripts was determined by quantitative RT-PCR (qPCR) from three separate pools of individuals at each developmental stage. The expression profile for each transcript is shown relative to the precompetent sample (sequencing, dashed blue lines; qPCR, solid red lines). The relative fold change in expression estimated by the two methods showed similar trends across development for 40 of the genes tested (Pearson r ≥ 0.70). Variance in pooled sample replicates was assessed using one-way ANOVA with Bonferroni’s post-test (***, p < 0.0001; **, p < 0.001; *, p < 0.01). Error bars indicate the standard deviation. Click here for file

Additional file 3 **Figure S3. Expression of annotated and non-annotated genes.** Sponge genes were aligned to sequences in the UniProt database. Sequences with significant matches (e-value ≤ 1x10^-4^) were designated as ‘annotated’ and those without as ‘non-annotated.’ (A) Non-annotated genes (red line) have lower overall expression compared to annotated genes (blue line). (B) Both gene sets exhibit similar patterns of variation across development. Heatmaps show relative expression of annotated and non-annotated genes (red, high; blue, low). The number of genes in each set is indicated to the left of each heatmap. Click here for file

Additional file 6 **Table S1. Four-fold differentially expressed genes at stage transitions.** List of genes that are differentially expressed (>4-fold and greater than sampling noise) at indicated stage transitions grouped by direction of change (up or downregulation). Transcript length, normalized read counts, name and accession number of best sequence match in the UniProt database, Gene Ontology (GO) annotation, PFAM domains, and PANTHER annotation is indicated for each gene. Click here for file

Additional file 7 **Table S2. Two-fold differentially expressed genes at stage transitions.** List of genes that are differentially expressed (>2-fold and greater than sampling noise) at indicated stage transitions grouped by direction of change (up or downregulation). Transcript length, normalized read counts, name and accession number of best sequence match in the UniProt database, Gene Ontology (GO) annotation, PFAM domains, and PANTHER annotation is indicated for each gene. Click here for file

Additional file 8 **Table S3. Gene ontology (GO) analysis for genes exhibiting greater than two- or four-fold change in expression between successive stages.** Selected functional terms enriched in the set of genes that are upregulated or downregulated at specific stage transitions are shown with the corresponding p-values. The number of genes belonging to each functional category in the genome or within each differentially expressed group is indicated. Click here for file

Additional file 9 **Table S4. PANTHER functional group enrichment analysis for genes that exhibit greater than four-fold change in expression between successive stages.** Functional groups enriched in the set of genes that are upregulated or downregulated between stages are shown (enrichment p-value ≤ 0.001). The number of genes in the genome or within each differentially expressed group that belong to a category is indicated. Click here for file

Additional file 4 **Figure S4. Expression of genes in selected functional groups.** Genes within each category were retrieved using the Gene Ontology (GO) annotation of their best UniProt sequence match or by the presence of PFAM domains. Transcription factors, G-protein coupled receptors, and kinase genes were obtained from previous studies [23,56,57]. Similar functional categories are shown together: (A) general cellular processes; (B) metabolic processes; (C) metazoa-associated processes; (D) regulators of gene expression; (E) transcription factors; (F) receptors and signaling mechanisms; (G) kinases. (Left) Percent of genes in each functional category that are detected by sequencing. The red and blue lines indicate the expected percent of genes to be found in at least one stage (45%) and at each stage (30%), respectively, based on the overall number of gene models detected by sequencing. The total number of predicted genes belonging to each category is shown. (Right) The percent of expressed genes in each functional category that are found within the top 25% of their expression range across the four developmental stages included in the study. Click here for file

Additional file 10 **Table S5. Genes exhibiting greater than 100-fold upregulation in relative expression level during sponge development.** Maximum upregulation (max. upreg.) is the highest relative expression ratio for the gene, given as the log_2_ of reads for a gene in one stage minus the average of the log_2_ of reads in the other three stages. Click here for file

Additional file 5 **Figure S5. Relative expression heatmaps for genes belonging to developmental signaling pathways, transcription factor families, or kinase classes.** (A) Genes in the Wnt, Notch, and TGF-β pathways have homologs in *A. queenslandica* (blue, present; gray, absent) and are expressed throughout sponge development (P, precompetent; C, competent; PL, postlarva; A, adult). Major receptors and ligands in each pathway are highlighted in red. (B) Relative expression of genes in transcription factor families. (C) Relative expression of genes in kinase classes. Heat maps show the relative expression of genes across stages (red, high; blue, low). Genes were retrieved from previous studies [10,11,23,87]. Stage enrichment of genes, based on the number that are found within the top 25% of their expression range across the four developmental stages, was estimated using Fisher’s exact test (p-values shown). Click here for file

Additional file 11 Table S6. Quantitative RT-PCR validation cycle numbers, read counts, and primer sequences. Click here for file
