# Supplementary figures and images for: Transcriptome profiling of the demosponge Amphimedon queenslandica reveals genome-wide events that accompany major life cycle transitions
Source: BMC Genomics. 2012 May 30;13:209. doi: 10.1186/1471-2164-13-209 (PMC3447736; doi:10.1186/1471-2164-13-209)

Figure S1

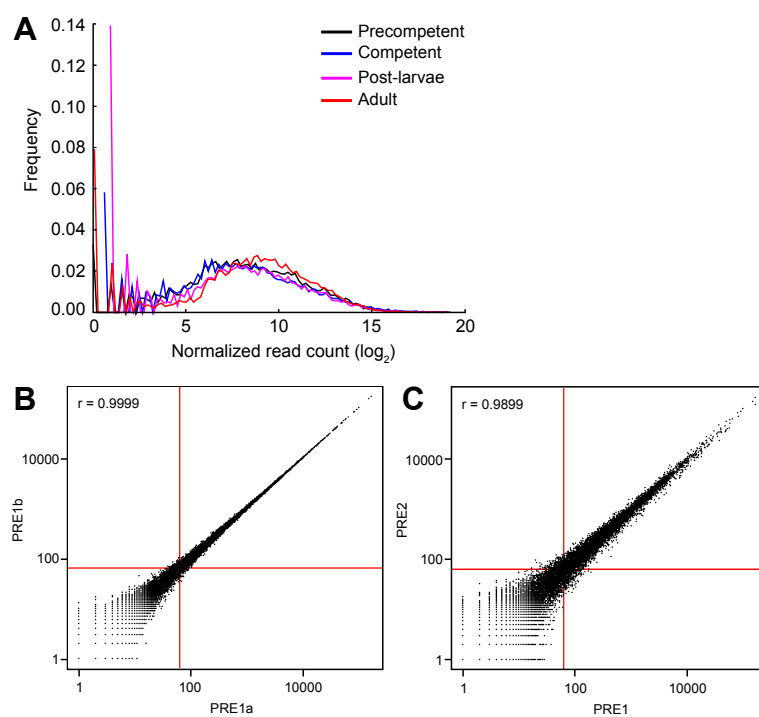

Supplement: Additional file 1 — Figure S1. Read normalization and setting the transcript detection threshold. (A) Distribution of transcript read counts after global normalization for sequencing depth. (B) Comparison of normalized read counts from two sequencing runs of the same library preparation and (C) comparison of normalized read counts from sequencing runs of two independent libraries made from the same RNA sample show that determination of transcript expression is reproducible above a cutoff threshold of 64 reads (red lines). [file 1471-2164-13-209-S1.pdf]

Figure S2

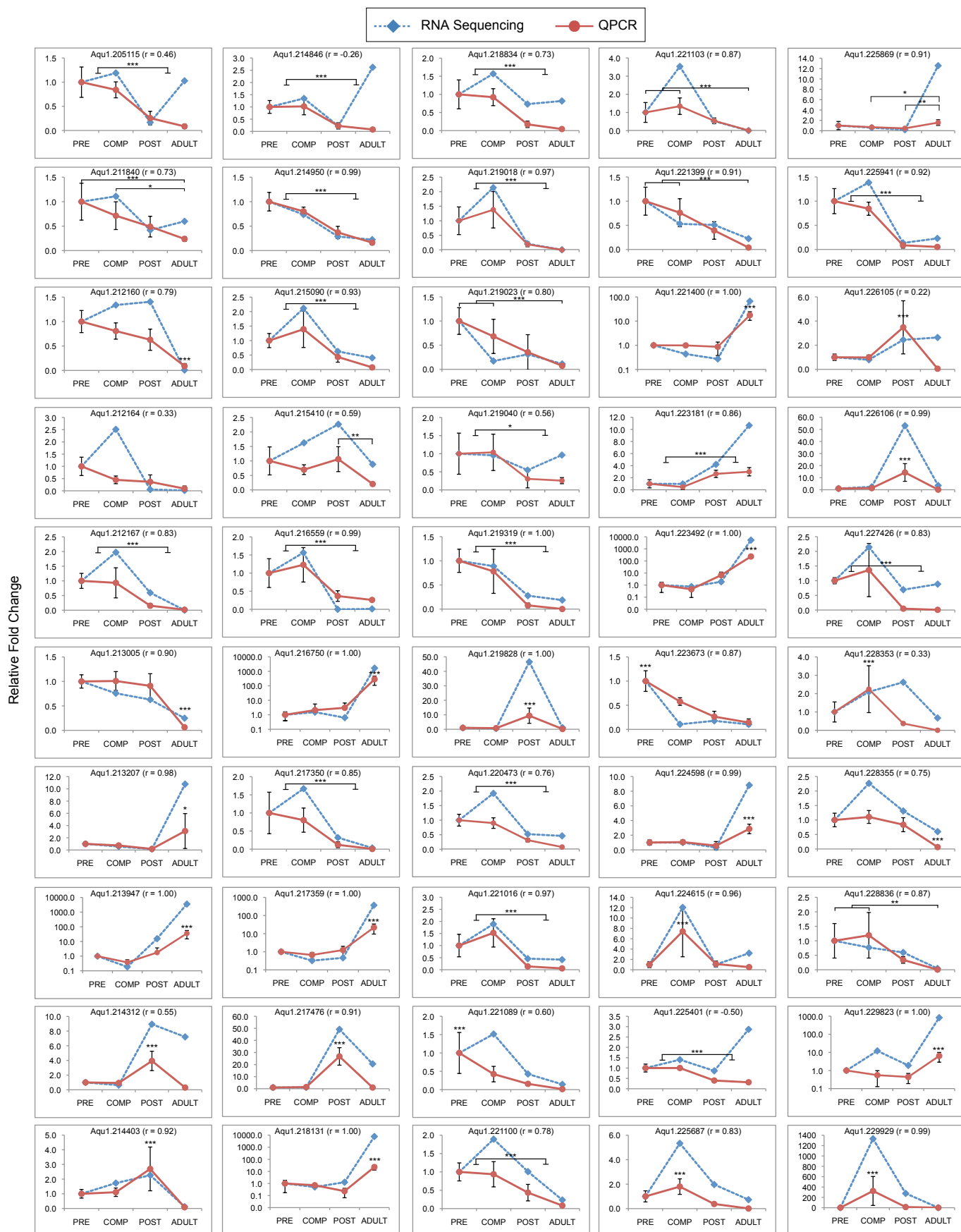

Supplement: Additional file 2 — Figure S2. Comparison of expression trends from sequencing and quantitative RT-PCR. The expression of 50 transcripts was determined by quantitative RT-PCR (qPCR) from three separate pools of individuals at each developmental stage. The expression profile for each transcript is shown relative to the precompetent sample (sequencing, dashed blue lines; qPCR, solid red lines). The relative fold change in expression estimated by the two methods showed similar trends across development for 40 of the genes tested (Pearson r ≥ 0.70). Variance in pooled sample replicates was assessed using one-way ANOVA with Bonferroni’s post-test (***, p < 0.0001; **, p < 0.001; *, p < 0.01). Error bars indicate the standard deviation. [file 1471-2164-13-209-S2.pdf]

Figure S3

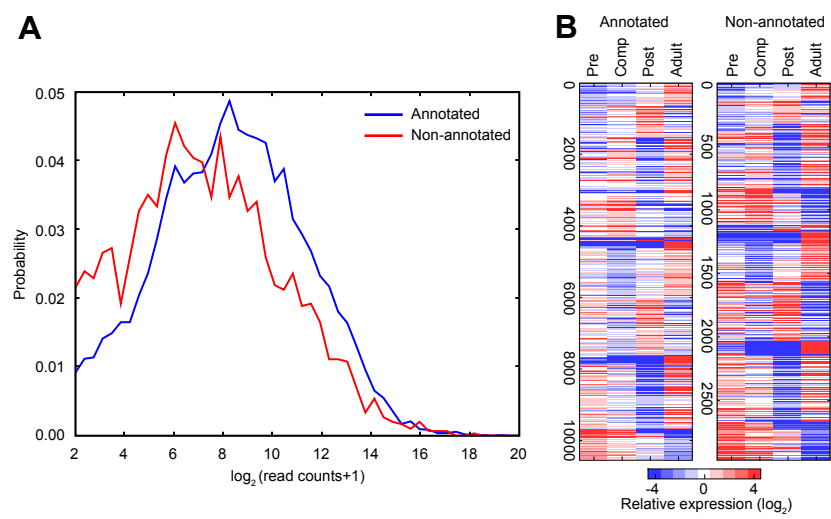

Supplement: Additional file 3 — Figure S3. Expression of annotated and non-annotated genes. Sponge genes were aligned to sequences in the UniProt database. Sequences with significant matches (e-value ≤ 1x10^-4^) were designated as ‘annotated’ and those without as ‘non-annotated.’ (A) Non-annotated genes (red line) have lower overall expression compared to annotated genes (blue line). (B) Both gene sets exhibit similar patterns of variation across development. Heatmaps show relative expression of annotated and non-annotated genes (red, high; blue, low). The number of genes in each set is indicated to the left of each heatmap. [file 1471-2164-13-209-S3.pdf]

Figure S4

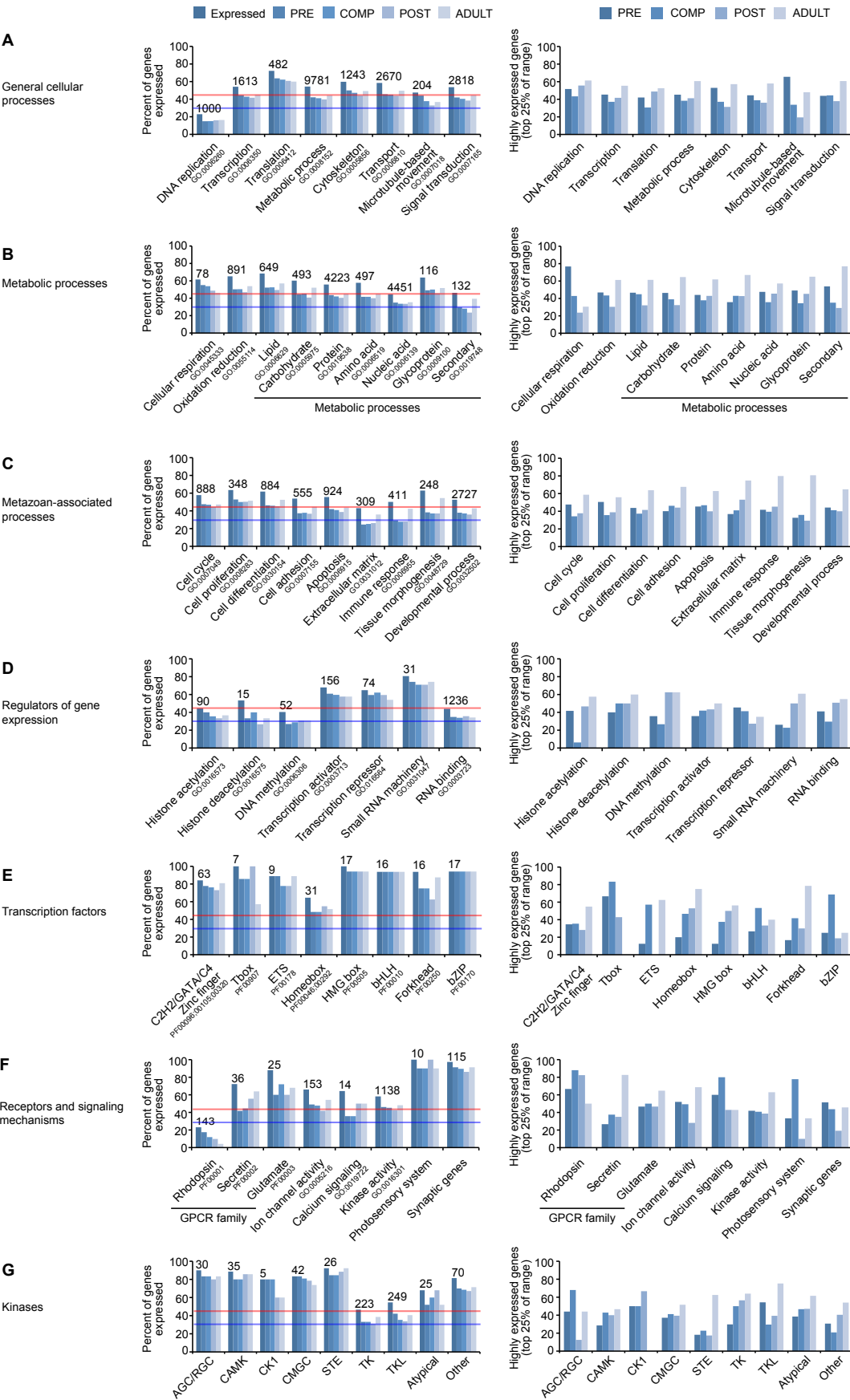

Supplement: Additional file 4 — Figure S4. Expression of genes in selected functional groups. Genes within each category were retrieved using the Gene Ontology (GO) annotation of their best UniProt sequence match or by the presence of PFAM domains. Transcription factors, G-protein coupled receptors, and kinase genes were obtained from previous studies [23,56,57]. Similar functional categories are shown together: (A) general cellular processes; (B) metabolic processes; (C) metazoa-associated processes; (D) regulators of gene expression; (E) transcription factors; (F) receptors and signaling mechanisms; (G) kinases. (Left) Percent of genes in each functional category that are detected by sequencing. The red and blue lines indicate the expected percent of genes to be found in at least one stage (45%) and at each stage (30%), respectively, based on the overall number of gene models detected by sequencing. The total number of predicted genes belonging to each category is shown. (Right) The percent of expressed genes in each functional category that are found within the top 25% of their expression range across the four developmental stages included in the study. [file 1471-2164-13-209-S4.pdf]

Figure S5

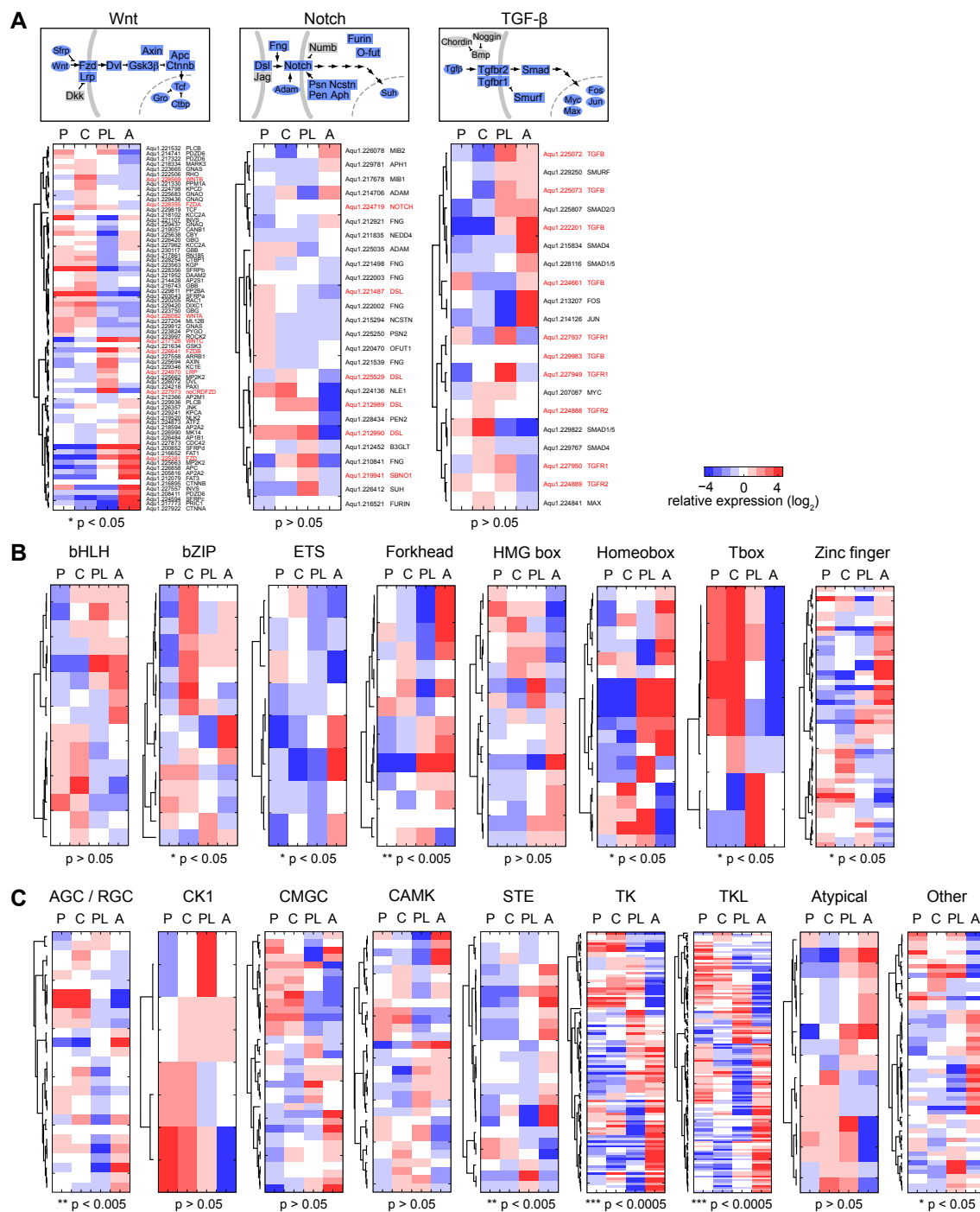

Supplement: Additional file 5 — Figure S5. Relative expression heatmaps for genes belonging to developmental signaling pathways, transcription factor families, or kinase classes. (A) Genes in the Wnt, Notch, and TGF-β pathways have homologs in A. queenslandica (blue, present; gray, absent) and are expressed throughout sponge development (P, precompetent; C, competent; PL, postlarva; A, adult). Major receptors and ligands in each pathway are highlighted in red. (B) Relative expression of genes in transcription factor families. (C) Relative expression of genes in kinase classes. Heat maps show the relative expression of genes across stages (red, high; blue, low). Genes were retrieved from previous studies [10,11,23,87]. Stage enrichment of genes, based on the number that are found within the top 25% of their expression range across the four developmental stages, was estimated using Fisher’s exact test (p-values shown). [file 1471-2164-13-209-S5.pdf]
